# O040: The effect of improved hand hygiene compliance on nosocomial transmission of Staphylococcus aureus

**DOI:** 10.1186/2047-2994-2-S1-O40

**Published:** 2013-06-20

**Authors:** V Weterings, JV Esser, RV Etten, J Kluytmans

**Affiliations:** 1 Microbiology and Infection control, the Netherlands; 2 Internal medicine, Amphia hospital, Breda, the Netherlands; 3 Microbiology and Infection control, VUMC, Amsterdam, the Netherlands

## Objectives

The objective of this prospective interventional study was to observe the effect of improved hand hygiene compliance(HHC) on nosocomial transmission of *S.aureus* between patients.

## Methods

The study was conducted between Oct 2011 and Dec 2012 in the oncology ward of a Dutch Teaching Hospital and contained multiple consecutive interventions: [I]increase number of hand alcohol dispensers; [II]education on HH; [III]replacing standard hand alcohol and soap dispensers by new automated dispensers, no feedback was given; [IV]personal feedback of HHC.

HHC was manually monitored according to the WHO method, twice a week during the whole study period. All patients were cultured weekly to detect nasal carriage of *S.aureus*. Isolates collected in period [II] and [IV] were typed using Amplification Fragment Length Polymorphism (AFLP). The ratio between secondary and primary cases, Transmission Index (TI), was calculated.

## Results

The HHC improved significantly from 31.5%(92/302;CI 25.3-36.0) in period [II] to 52.9%(139/263;CI 46.6-59.0) in period [IV](p<0.001).

In period [II] 266 patients were hospitalized on the days of culture; 246 nasal swabs(92.5%) were collected from 151 unique patients. In total 42/151 patients(27.5%) were *S.aureus* carriers. AFLP revealed 6 unrelated isolates and 13 clusters (2-14 isolates,median 3). Number of primary cases(PC) was 19. Transmission of *S.aureus* from a PC to other patients occurred in 10 out of 19(52.6%) PC, resulting in 22 secondary cases(SC). TI of 1.2(22/19).

In period [IV] 314 patients were hospitalized on the days of culture; 268 nasal swabs(85.4%) were collected from 134 unique patients. In total 45/134 patients(33.6%) were *S.aureus* carriers. AFLP revealed 16 unrelated isolates and 9 clusters (2-7 isolates,median 3). Number of PC was 25. Transmission of *S.aureus* from a PC to other patients occurred in 9 of 25(36.0%) PC, resulting in 17 SC. TI of 0.7(17/25).

The ratio of unique versus clustered strains was significantly higher in period IV(p=0.028).

## Conclusion

An improvement of HHC from 31.5% to 52.9%(RR:1.68) was associated with a 32%reduction of the TI. This study shows that improvement of HHC using automatic dispensers with personal feedback reduces the transmission of *S.aureus* in the hospital substantially.

## Disclosure of interest

None declared.

